# Indole-based hybrids target both asexual parasites and gametocytes of *Plasmodium falciparum* and synergize with lumefantrine

**DOI:** 10.1128/aac.00490-26

**Published:** 2026-07-01

**Authors:** Abhinab Mohanty, Myna Nakabashi, Barbara K. M. Dias, Pedro Maiolini, José Antonio Campos Delgado, Karina de Souza Quaglio, Márcio Weber Paixão, Celia R. S. Garcia

**Affiliations:** 1 Department of Clinical and Toxicological Analyses, School of Pharmaceutical Sciences, University of São Paulo 505153 , São Paulo, São Paulo, Brazil; 2 Laboratory for Sustainable Organic Synthesis and Catalysis, Department of Chemistry, Federal University of São Carlos–UFSCar 201361, São Carlos, São Paulo, Brazil; The Children's Hospital of Philadelphia, Philadelphia, Pennsylvania, USA

**Keywords:** gametocyte, indole derivatives, antimalarials, *Plasmodium falciparum*, combination therapy

## Abstract

Malaria remains a persistent global health challenge, and emerging resistance to artemisinin-based therapy threatens disease control. Here, we report the evaluation of scaffolds selected from three classes of indole hybrids. A library of 24 synthetically designed indoles was screened *in vitro* against *Plasmodium falciparum* sexual and asexual forms. The derivatives displayed a wide range of activity, from inactive to potent, with compound ARN53 exhibiting the strongest inhibition (IC₅₀ = 684.8 ± 289.3 nM). Cytotoxicity assays in HEK293T mammalian cells demonstrated the active compounds to be more selective toward the malaria parasite. Remarkably, the potent analogs retained efficacy against the multidrug-resistant Dd2 strain, suggesting a capacity to overcome established resistance mechanisms. Interestingly, ARN53 displayed detectable activity against sexual stages and demonstrated a synergistic effect with lumefantrine, supporting its potential for combination therapy. Overall, these findings expand the chemical diversity of indole scaffolds and highlight ARN53 and its analogs as promising leads for further optimization in antimalarial drug discovery.

## INTRODUCTION

Malaria, caused by *Plasmodium* parasites, remains a significant global health burden, particularly in tropical regions, affecting millions annually (1, 2). The emergence of resistance to first-line antimalarials highlights the urgent need for novel drugs with distinct mechanisms of action (3, 4). The World Health Organization (WHO) currently recommends artemisinin-based combination therapies (ACTs), which pair artemisinin or its derivatives with a longer-acting partner drug (5–7). Among these, the development of triple artemisinin-based combination therapies (TACTs), which combine an artemisinin derivative with two partner drugs, has been gaining increasing attention in the current malaria elimination landscape (8, 9). However, increasing reports of partial artemisinin resistance (10) are alarming, and the clinical development of TACT has proven challenging (11), reinforcing the need for continuous surveillance and drug discovery efforts. Monitoring *Plasmodium falciparum* resistance patterns and understanding their molecular basis are therefore critical not only for guiding therapeutic policy but also for shaping rational drug design strategies to circumvent cross-resistance mechanisms (12, 13).

A deeper understanding of parasite biology is key to identifying novel therapeutic targets. *Plasmodium* species typically exhibit synchronous development within the host, leading to the simultaneous release of merozoites into the bloodstream (14), thereby overwhelming the immune system (15). Studies by Hotta et al. (16) revealed that the host hormone melatonin regulates this synchronization in *Plasmodium chabaudi*, a rodent strain. In pinealectomized mice lacking melatonin, parasites lose synchrony, leading to an increase in mature intraerythrocytic forms. Melatonin treatment in *P. falciparum* cultures altered parasite development, leading to a greater proportion of schizont-stage parasites *in vitro* (16). These findings established the first experimental link between host hormonal signaling and the timing of parasite development.

Melatonin’s effects are mediated through a signaling cascade involving phospholipase C (PLC) activation, diacylglycerol (DAG), and inositol trisphosphate (IP3) production, and calcium release from the endoplasmic reticulum (17, 18). Key downstream effectors include *P. falciparum* atypical protein kinase 7 (PfPK7) (19), PfPKA (17, 20), PfeIK1 (21), and the nuclear protein PfMORC (22). Additionally, melatonin influences mitochondrial fission (23) and the ubiquitin-proteasome system (19, 24), further modulating parasite development.

Given melatonin’s role in parasite synchronization (25), indole-based compounds have been explored to disrupt the *Plasmodium* cell cycle. Structure–activity relationship (SAR) studies demonstrate that substitution patterns on the indole scaffold, particularly the presence of halogens and alkyl groups, influence antiplasmodial activity and interactions with parasite targets (26, 27) while also affecting properties related to bioavailability. This supports the indole framework as a basis for multitarget antiparasitic design (28). Consistent with this, indolyl-3-ethanone ethers and thioethers have contributed to SAR development (29), and indole derivatives targeting the melatonin pathway in *Plasmodium falciparum* interfere with parasite circadian regulation (30). In line with these observations, 2-sulfenylindole derivatives modulate parasite rhythms via melatonin-dependent pathways (31). Meanwhile, melatonin-derived 1H-1,2,3-triazole and benzene derivatives exhibit activity against asexual stages and late-stage gametocytes, with some compounds acting as agonists or antagonists of the parasite melatonin pathway (32).

The therapeutic potential of indole-based compounds extends beyond malaria to other parasitic diseases, including trypanosomiasis and leishmaniasis, with their versatility linked to their ability to disrupt key parasite biological pathways (33). Synthesized indoles in the form of marinoquinolines have demonstrated efficacy against *Toxoplasma gondii* (34), while newly developed formyl indole-based compounds show promising activity against *Cryptosporidium* infections (35). Notably, microbiota-derived indole has also been reported to inhibit *Cryptosporidium parvum* growth (36) and may serve as a biomarker of host susceptibility to infection (37).

These findings highlight the growing significance of indole-based compounds in antimalarial drug development. Their structural versatility, ability to target multiple biological pathways, and promising safety and efficacy make them strong candidates for further development. In this study, we report the evaluation of 24 modified indole hybrids, organized into three different structural series, against *Plasmodium falciparum*: 16 hybrids belong to the series of noncanonical, protected C-2 peripherally modified tryptophan-containing peptides; five hybrids belong to a series of canonical, unprotected tryptophan-containing peptides; and three compounds are built on indole-fused heterocyclic scaffolds. By integrating structure-based optimization with biological screening, this work seeks to expand the accessible chemical space of indole scaffolds and identify potent, selective candidates suitable for combination therapy to combat emerging drug resistance.

## MATERIALS AND METHODS

### *Plasmodium* and mammalian cell culture

The *Plasmodium falciparum* strains 3D7, Dd2, and NF54-GFP-luc were cultured in 175 cm² flasks (Greiner Bio-One) using RPMI 1640 medium containing 25 mM HEPES, 0.23% sodium bicarbonate, and 1% D-glucose. The 3D7 and Dd2 strains were maintained in medium supplemented with 0.5% Albumax I, while the modified NF54 strain was grown in medium enriched with 10% heat-inactivated human serum. Parasites were kept in human red blood cells at approximately 3% hematocrit and incubated at 37°C. Cultures were gassed with a 5% CO₂, 3% O₂, and 92% N₂ mixture for 30 s and then maintained at 37°C. The development of parasites was monitored daily using Panoptico-stained smears.

Human embryonic kidney (HEK293T) cells were cultured in 75 cm² flasks (Greiner Bio-One) at 37°C with 5% CO₂. Cells were maintained in Dulbecco’s Minimum Essential Medium (DMEM) supplemented with 10% fetal bovine serum (FBS), 3.7 g/L NaHCO₃ (Sigma), 100 U/mL penicillin, and 100 μg/mL streptomycin. The confluency of the cell culture was monitored visually under a microscope.

The *P. falciparum* 3D7 and Dd2 strains were used to evaluate antiplasmodial activity against asexual stages, while the NF54-GFP-luc strain was utilized for gametocyte production to conduct gametocytocidal experiments. The HEK293T cells were used to evaluate the cytotoxicity of compounds showing promising antimalarial activity.

### Flow cytometry for antimalarial assessment in *P. falciparum*

To determine the half-maximal inhibitory concentration (IC₅₀) of indole derivatives against the asexual stages of *Plasmodium falciparum* 3D7 and Dd2 strains, infected red blood cells (iRBCs) from an asynchronous culture were used. The assay was conducted in 96-well U-bottom plates at an initial parasitemia of 0.3% and a hematocrit of 1%. The iRBCs were incubated with serially diluted concentrations of the test compounds, depending on their maximum solubility (5–100 µM) in a final volume of 200 µL per well of complete RPMI 1640 media for 72 h at 37°C under a controlled gas mixture (5% CO₂, 3% O₂, and 92% N₂). A solvent control group was treated with an equivalent volume of dimethyl sulfoxide (DMSO) to account for any solvent-related effects.

Following incubation, iRBCs were stained with 1× SYBR Green I (Invitrogen) to label nucleic acids and 50 nM MitoTracker Deep Red (Invitrogen) to assess mitochondrial membrane potential and viability. Stained cells were incubated for 20 min at 37°C before analysis by flow cytometry using a BD Accuri C6 Flow Cytometer, with a collection threshold of 10⁴ events. Parasitemia was quantified through dot plot analysis using FlowJo 5 software. IC₅₀ values were calculated using GraphPad Prism 8, based on data from at least three independent experiments performed in triplicate.

### Cytotoxicity assay in HEK293T cells

To evaluate the cytotoxicity of the test compounds in mammalian cells, HEK293T cells were seeded in flat-bottom 96-well plates at a density of 10⁴ cells per well and incubated for 24 h in 100 µL of complete DMEM medium at 37°C with 5% CO₂. Subsequently, cells were treated with varying concentrations of each compound (0–100 µM) in 200 µL of complete DMEM medium and incubated for an additional 72 h under the same conditions. Control wells received DMSO at the highest solvent concentration used in the treatments.

Following incubation, 40 µL of MTT reagent (5 mg/mL in PBS) was added to each well, and plates were incubated for 3 h at 37°C with 5% CO₂. The media was then removed, and 100 µL of DMSO was added to each well to dissolve the formazan crystals. Plates were agitated for 10 min before measuring absorbance at 570 nm using a FlexStation 3 microplate reader (Molecular Devices). Three independent experiments, performed in triplicate, were used to calculate the half-maximal effective concentration (CC_50_) using GraphPad Prism 8.

### Luciferase assay for evaluating gametocytocidal activity in *P. falciparum* NF54-GFP-luc

Late-stage gametocytes (stages IV–V) from the *Plasmodium falciparum* NF54-GFP-luc line were enriched using magnetic cell separation (Miltenyi VarioMACS). Gametocytes were seeded in 96-well plates at 1% gametocytemia and 2% hematocrit in complete RPMI 1640 medium supplemented with 10% inactivated human serum.

Test compounds were serially diluted in fivefold steps and added to the cultures, which were then incubated under hypoxic conditions at 37°C for 72 h. Following incubation, luciferase activity was quantified as a measure of gametocyte viability. For this assay, the parasites were lysed, and 20 μL of parasite lysate was mixed with 20 μL of luciferin substrate (Promega Luciferase Assay System) at room temperature. Bioluminescence was measured over a 10-second integration period using the Berthold Tristar 5 Multi-Detection System. Gametocytocidal activity was determined by calculating the inverse of survival, as inferred from luminescence values. Three independent experiments were performed in triplicate, and the percent gametocytocidal activity was plotted using GraphPad Prism 8.

### Combination assay through modified fixed-ratio isobologram method

To evaluate the combinatorial effects of ARN53 with the established antimalarials chloroquine, primaquine, and lumefantrine, a modified fixed-ratio isobologram method was employed (38–40). For each drug, the top concentration was set at eightfold above its respective IC₅₀ value so that the IC₅₀ in this assay would fall near the midpoint of a twofold serial dilution, carried out six times. *P. falciparum* 3D7 parasites were incubated with the drug combinations in 96-well U-bottom plates at an initial parasitemia of 0.3% and hematocrit of 1% for 72 h. The drugs were tested at fixed ratios of 5:0, 4:1, 3:2, 1:1, 2:3, 1:4, and 0:5. IC₅₀ values were determined using the double-staining method described above.

The half-maximal fractional inhibitory concentration (FIC₅₀) was calculated as the ratio of the IC₅₀ of a drug in combination to the IC₅₀ of the drug alone. The sum FIC₅₀ (ΣFIC₅₀) was obtained as follows:

∑FIC_50_ = (IC_50_ of Drug A in combination/ IC_50_ of Drug A alone) + (IC_50_ of Drug B in combination/ IC_50_ of Drug B alone)

Isobologram curves were generated by plotting FIC₅₀A vs FIC₅₀B. A straight diagonal line (ΣFIC₅₀ ≈ 1) indicates an additive effect (no interaction), a concave curve below the diagonal (ΣFIC₅₀ <1) indicates synergism, and a convex curve above the diagonal (ΣFIC₅₀ >1) indicates antagonism. All statistical analyses and isobologram plotting were performed using GraphPad Prism 8.

### “Indole-cored” fused compounds

Compounds evaluated in this study were synthesized following standard chemical procedures reported elsewhere. Accordingly, supplementary material for synthesis and the characterization data for noncanonical, protected C-2 peripherally modified tryptophan-containing peptides can be found in reference 41, for canonical, unprotected tryptophan-containing peptides are found in reference 42, and for the indole-fused heterocyclic scaffolds are found in reference 43.

## RESULTS

### Indole compounds exhibit varied potency against asexual stages of *P. falciparum*

**Fig 1:**
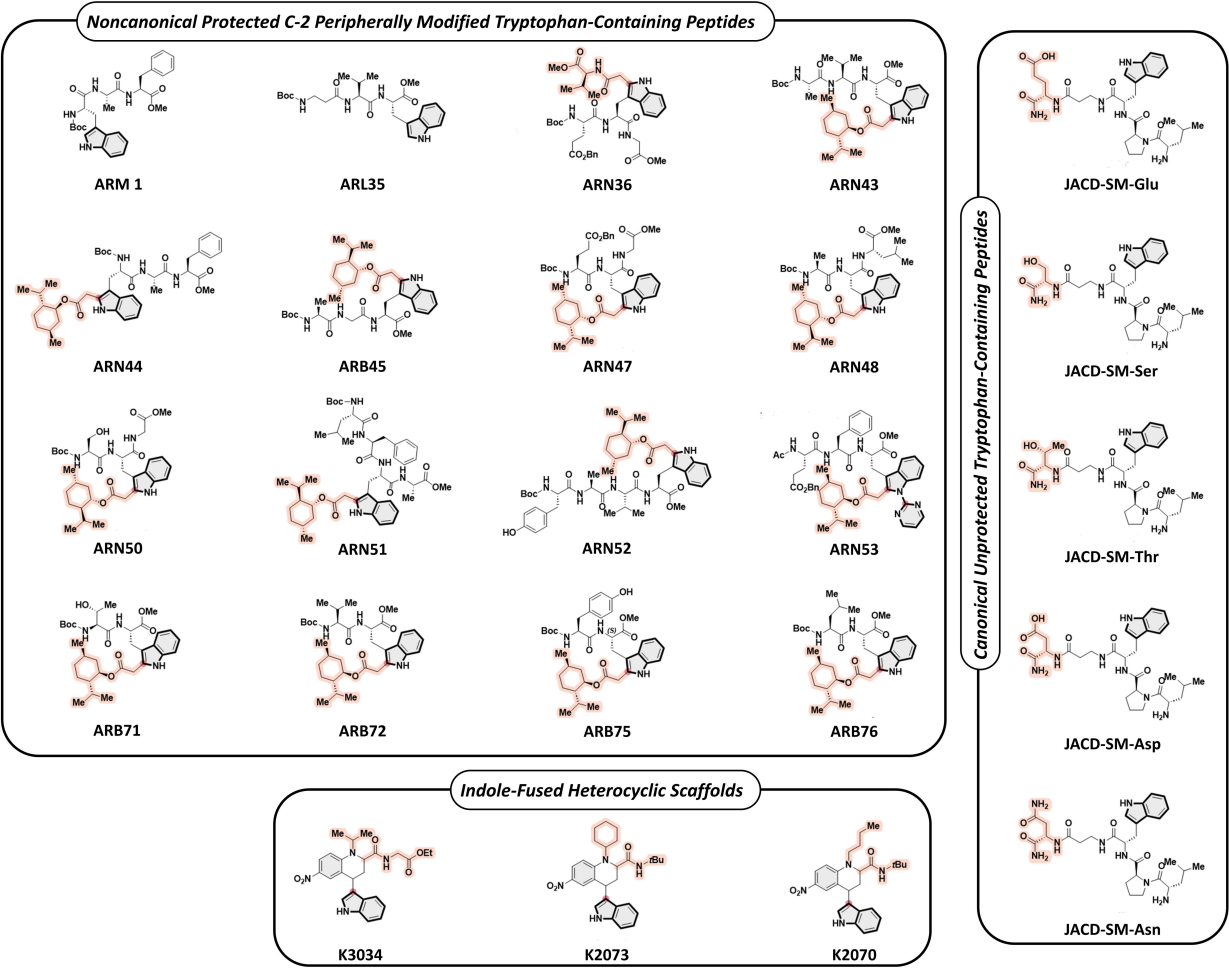
Chemical structures of hybrid indole-based compounds evaluated in this study. The structures comprise a curated library of 24 hybrid molecules spanning three distinct structural classes designed to probe chemical space around the indole scaffold. The largest subset comprises 16 noncanonical, protected C-2–functionalized tryptophan-derived peptides. The second group includes five canonical, unprotected tryptophan-containing peptides that preserve the native indole framework while varying the surrounding amino acid environment. The third class comprises three indole-fused heterocyclic scaffolds representing more structurally constrained architectures. Together, this collection captures a range of steric, electronic, and conformational features, enabling assessment of how indole functionalization and scaffold context influence biological activity.

The antimalarial activity of 24 indole compounds (Fig. 1) was evaluated *in vitro* against the asynchronous culture of wild-type *Plasmodium falciparum* (3D7). To determine the IC₅₀ values, parasites were exposed to serially diluted concentrations of the test compounds, depending on their maximum solubility (5–100 µM), for 72 h. Final parasitemia was assessed by flow cytometry using a double-staining method. Nucleic acid labeling was performed using SYBR Green I, and MitoTracker Deep Red was used to selectively stain mitochondria in live, healthy parasites.

**Fig 2:**
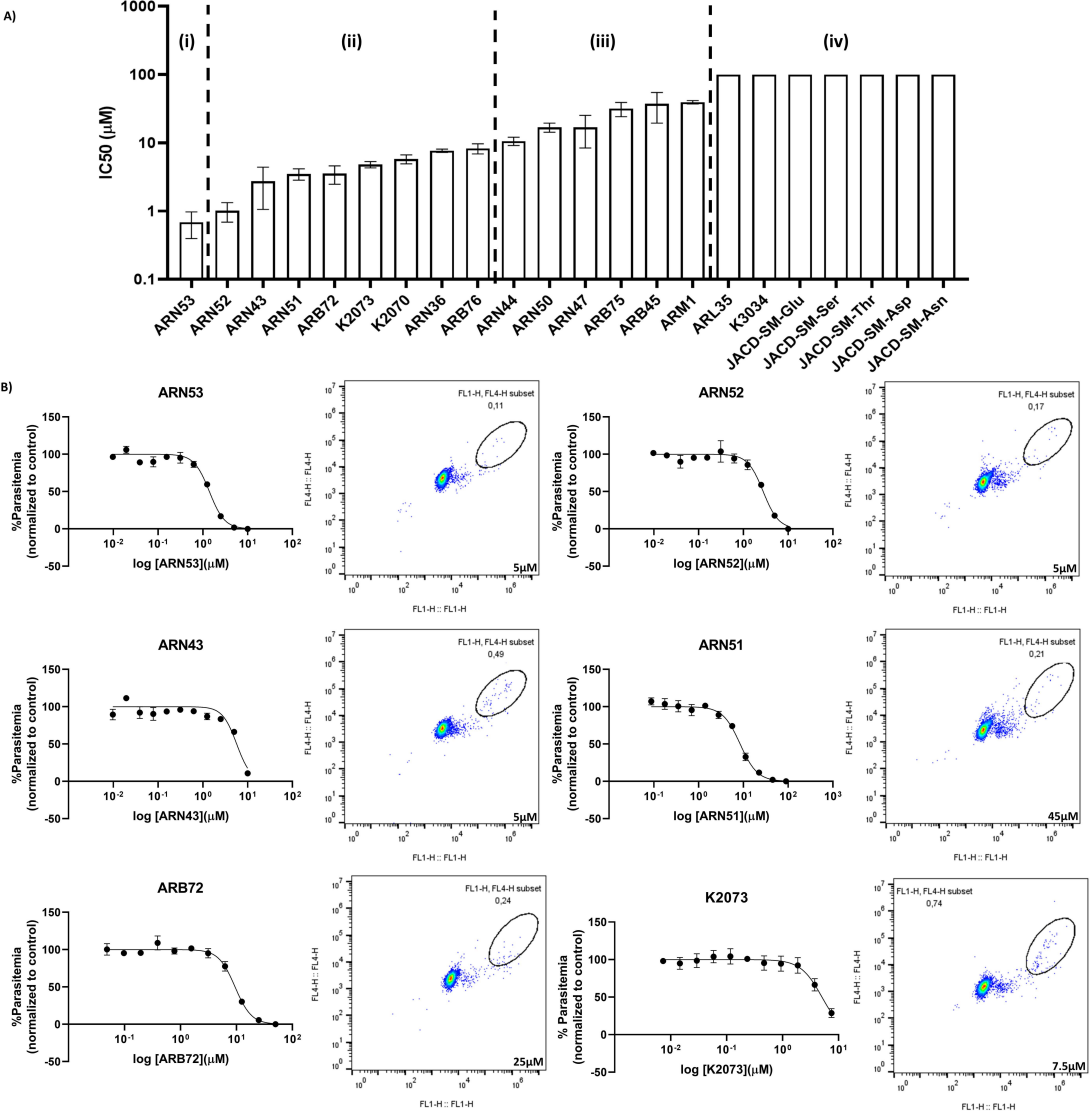
Antiplasmodial activity of hybrid compounds against *P. falciparum* 3D7 parasites. (**A**) Histogram depicting the distribution of IC₅₀ values for all compounds tested, grouped according to activity. Compounds are classified as (i) potent, displaying nanomolar values in the series; (ii) moderately active, with IC₅₀ values in the low micromolar range (<10 µM); (iii) weakly active, showing inhibitory effects only at higher micromolar concentrations (>10 µM); and (iv) inactive, with no detectable activity within the tested concentration range. This classification highlights the spread of activities across the library and facilitates comparison of performance. (**B**) Representative curves for the best six compounds (ARN53, ARN52, ARN43, ARN51, ARB72, and K2073) following 72 h of treatment, with parasitemia normalized to DMSO-treated controls. The sigmoidal curves illustrate concentration-dependent inhibition of parasite growth. The corresponding flow cytometry dot plots show the distribution of infected erythrocytes at the indicated highest concentration. Data are presented as mean ± S.D. from three independent experiments, each performed in triplicate.

A myriad of antimalarial responses was observed among the 24 indole compounds evaluated against the parasite (Table 1). Based on their activity, the compounds were categorized into four groups (Fig. 2). The first group consisted of inactive compounds that did not exhibit detectable antimalarial activity within the tested concentration range. ARL35 (IC₅₀: 185.9 ± 32.15 µM), JACD-SM-Glu, JACD-SM-Ser, JACD-SM-Thr, JACD-SM-Asp, JACD-SM-Asn, and K3034 fell into this category. The second group included weakly active compounds that demonstrated minimal activity, with inhibitory effects observed only at high micromolar concentrations (IC₅₀ >10 µM). Compounds ARN44 (IC₅₀: 10.59 ± 1.47 µM), ARN50 (IC₅₀: 16.84 ± 2.55 µM), ARN47 (16.84 ± 8.42 µM), ARB75 (IC₅₀: 31.61 ± 7.41 µM), ARB45 (37.07 ± 17.65 µM), and ARM1 (39.32 ± 2.2 µM) were classified under this category. The third group comprised moderately active compounds that exhibited improved efficacy, with IC₅₀ values in the lower micromolar range (IC₅₀ <10 µM). Compounds ARN52 (IC₅₀: 1.01 ± 0.32 µM), ARN51 (IC₅₀: 3.5 ± 0.65 µM), ARB72 (IC₅₀: 3.53 ± 1.06 µM), ARN36 (7.66 ± 0.42 µM), ARB76 (IC₅₀: 8.267 ± 1.41 µM), ARN43 (IC₅₀: 2.73 ± 1.67 µM), K2070 (IC_50_: 5.78 ± 0.86 µM), and K2073 (IC_50_: 4.82 ± 0.51 µM) demonstrated modest activity. The fourth and most promising group included highly active compounds, with ARN53 (IC₅₀: 0.6848 ± 0.2893 µM) emerging as the most potent, having a half-maximal inhibitory concentration of approximately 685 nM. The representative IC_50_ curves for all the compounds are available in Fig. S1.

**TABLE 1:** Activity of indole-derived compounds and reference drugs against *Plasmodium falciparum* 3D7^*a*^

Compound	IC_50_ ± S.D.	Compound	IC_50_ ± S.D.
ARM1	39.32 ± 2.2 µM	ARB75	31.61 ± 7.41 µM
ARL35	185.9 ± 32.15 µM	ARB76	8.27 ± 1.41 µM
ARN36	7.66 ± 0.42 µM	JACD-SM-Glu	N.D.
ARN43	2.73 ± 1.67 µM	JACD-SM-Ser	N.D.
ARN44	10.59 ± 1.47 µM	JACD-SM-Thr	N.D.
ARB45	37.07 ± 17.65 µM	JACD-SM-Asp	N.D.
ARN47	16.84 ± 8.42 µM	JACD-SM-Asn	N.D.
ARN48	N.S.	K3034	N.D.
ARN50	16.84 ± 2.55 µM	K2073	4.82 ± 0.51 µM
ARN51	3.5 ± 0.65 µM	K2070	5.78 ± 0.86 µM
ARN52	1.01 ± 0.32 µM	CQ	18.16 ± 2.07 nM
ARN53	0.6848 ± 0.2893 µM	PMQ	275.3 ± 34.56 nM
ARB71	N.S.	LF	3.259 ± 1.86 nM
ARB72	3.53 ± 1.06 µM		

^*a*^ Half-maximal inhibitory concentrations (IC₅₀) were determined using *in vitro* growth inhibition assays and are reported as mean ± standard deviation (S.D.) (µM or nM) from independent experiments performed in triplicate. CQ, chloroquine; PMQ, primaquine; LF, lumefantrine. N.D., not determined; N.S., not soluble in RPMI culture medium.

### Indole compounds possess low toxicity to mammalian cells

To determine whether the active compounds were toxic to mammalian host cells, we next evaluated their cytotoxic potential. This was an important step to ensure that the activity we observed was selective for the parasite and not due to general toxicity. For this purpose, we employed the MTT assay using HEK293T cells as a representative mammalian cell line.

**Fig 3:**
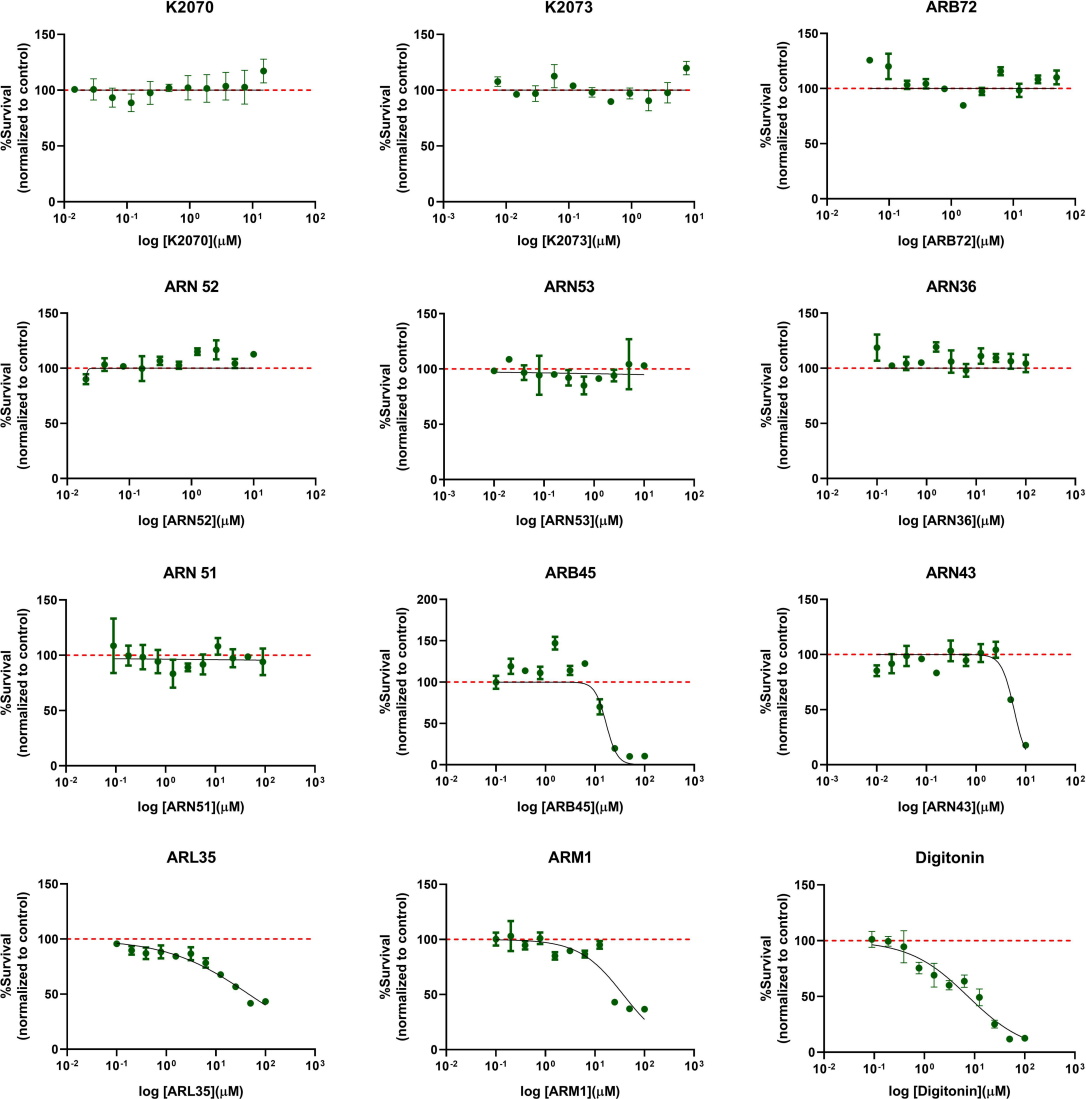
Cytotoxicity profiles of hybrid compounds against HEK293T mammalian cells. Survival curves for selected compounds (K2070, K2073, ARB72, ARN52, ARN53, ARN51, ARB45, ARN36, ARN43, ARL35, and ARM1) were generated using an MTT-based viability assay in HEK293T cells, with digitonin included as a positive control. Cells were exposed to increasing concentrations of each compound, and viability was assessed after incubation under standard culture conditions for 72 h. Cell viability was normalized to the DMSO-treated controls and expressed as a percentage of survival. Most compounds showed minimal cytotoxicity across the tested concentration range, whereas ARB45, ARL35, and ARM1 displayed a dose-dependent reduction in cell viability, consistent with the effect observed for digitonin. The survival curves presented are representative of three independent experiments.

Of the 11 compounds tested, four exhibited cytotoxic effects at micromolar concentrations (Fig. 3). Specifically, ARM1 showed toxicity at 38.78 µM, ARB45 at 77.62 µM, ARL35 at 40.94 µM, and ARN43 at a notably lower concentration of 4.69 µM, indicating stronger cytotoxicity. In contrast, the remaining seven compounds—ARN36, ARN52, ARN51, ARB72, ARN53, K2070, and K2073—did not exhibit any detectable toxicity under the same conditions. Remarkably, all of these non-cytotoxic compounds also retained antiplasmodial activity, with IC₅₀ values below 10 µM.

Selectivity analysis supports the therapeutic potential of this series (Table 2). Non-cytotoxic compounds showed favorable selectivity, with ARN36 and ARN51 exhibiting the highest SI values (≥13.05 and ≥12.86), while ARN53 and ARB72 displayed moderately lower-bound selectivity estimates, which may be attributed to limitations in aqueous solubility. In contrast, compounds with measurable cytotoxicity (ARN43, ARL35, ARM1, and ARB45) showed low SI values, indicating limited parasite selectivity.

**TABLE 2:** Cytotoxicity and selectivity index (SI) of selected compounds against mammalian HEK293T cells^*a*^

Compound	CC_50_ ± S.D.	S.I. (CC_50_/IC_50_^3D7^)
K2070	≥15 µM	≥2.59
K2073	≥7.5 µM	≥1.56
ARB72	≥25 µM	≥7.08
ARN52	≥5 µM	≥4.95
ARN53	≥5 µM	≥7.30
ARN36	≥100 µM	≥13.05
ARN51	≥45 µM	≥12.86
ARB45	72.62 ± 65.61 µM	1.96
ARN43	4.7 ± 1.12 µM	1.72
ARL35	40.94 ± 14.36 µM	0.22
ARM1	38.78 ± 18.06 µM	0.99
DG	46.13 ± 55.01 µM	N.D.

^*a*^ Half-maximal cytotoxic concentrations (CC₅₀) were determined using an MTT-based viability assay and are presented as mean ± standard deviation (S.D.) from three independent experiments performed in triplicate. The selectivity index (S.I.) was calculated as the ratio of CC₅₀ to IC₅₀ obtained against *Plasmodium falciparum* 3D7 parasites (SI = CC₅₀ / IC₅₀^3D7^), representing the parasite selectivity of the compound over the host cell. For compounds where no cytotoxicity was observed within the tested concentration range, CC₅₀ values are reported as greater than or equal to (≥) the highest concentration tested, and corresponding SI values represent the lower-bound estimates. N.D., not determined.

These findings are highly encouraging, as they suggest that most of the active compounds in our study exhibit high parasite selectivity. The seven non-toxic compounds, in particular, stand out as promising scaffolds that could be further refined and optimized for improved potency and drug-like properties. Overall, with only four out of 11 active compounds exhibiting cytotoxicity, our results suggest that most members of this chemical series are well tolerated by mammalian cells and hold significant potential for further preclinical development.

### Differential activity observed in multidrug-resistant parasite

We further evaluated the seven non-toxic compounds for their efficacy against the multidrug-resistant *P. falciparum* strain Dd2. The Dd2 strain was originally isolated in the Indochina region and exhibits resistance to chloroquine, mefloquine, sulfadoxine, and pyrimethamine (44–46). Chloroquine remains one of the most commonly used antimalarial drugs worldwide, while sulfadoxine-pyrimethamine (SP) remains a chemopreventive treatment for *Plasmodium falciparum*, particularly in pregnant women, infants, and children (47). However, growing resistance to these important drugs continues to threaten effective malaria treatment and prevention. Identifying compounds that can bypass or overcome such resistance mechanisms is therefore a critical step in the drug discovery process.

The seven compounds were tested against the Dd2 strain, and their IC₅₀ values were compared with those obtained for the sensitive 3D7 strain (Table 3). This comparison allowed us to determine whether any of the compounds retained or even improved their activity in the presence of the resistance mechanism or, conversely, whether their efficacy was compromised.

**TABLE 3:** Activity of compounds with no toxicity against the drug-resistant *Plasmodium falciparum* Dd2 strain and resistance index (RI)^*a*^

Compound	IC_50_^Dd2^ ± S.D.	R.I. (IC_50_^Dd2^/IC_50_^3D7^)
ARB72	4.03 ± 0.42 µM	1.14
ARN53	0.785 ± 0.064 µM	1.15
ARN51	2.77 ± 0.35 µM	0.79
ARN52	1.77 ± 0.2 µM	1.75
K2070	7.04 ± 0.71 µM	1.22
K2073	4.37 ± 0.6 µM	0.91
ARN36	42.89 ± 1.42 µM	5.6
CQ	86.92 ± 31.46 nM	4.8

^*a*^ Half-maximal inhibitory concentrations (IC₅₀) against the Dd2 strain were determined using *in vitro* asexual blood-stage growth inhibition assays and are presented as mean ± standard deviation (S.D.) from three independent experiments performed in triplicate. The resistance index (R.I.) was calculated as the ratio of IC₅₀ values obtained for the resistant Dd2 strain relative to the drug-sensitive 3D7 strain (R.I. = IC₅₀^Dd2^ / IC₅₀^3D7^). R.I. values ≈1 indicate comparable activity, values <1 indicate increased potency towards Dd2, values >1 indicate a reduction in potency, and values >5 mark resistance in Dd2. CQ = Chloroquine.

**Fig 4:**
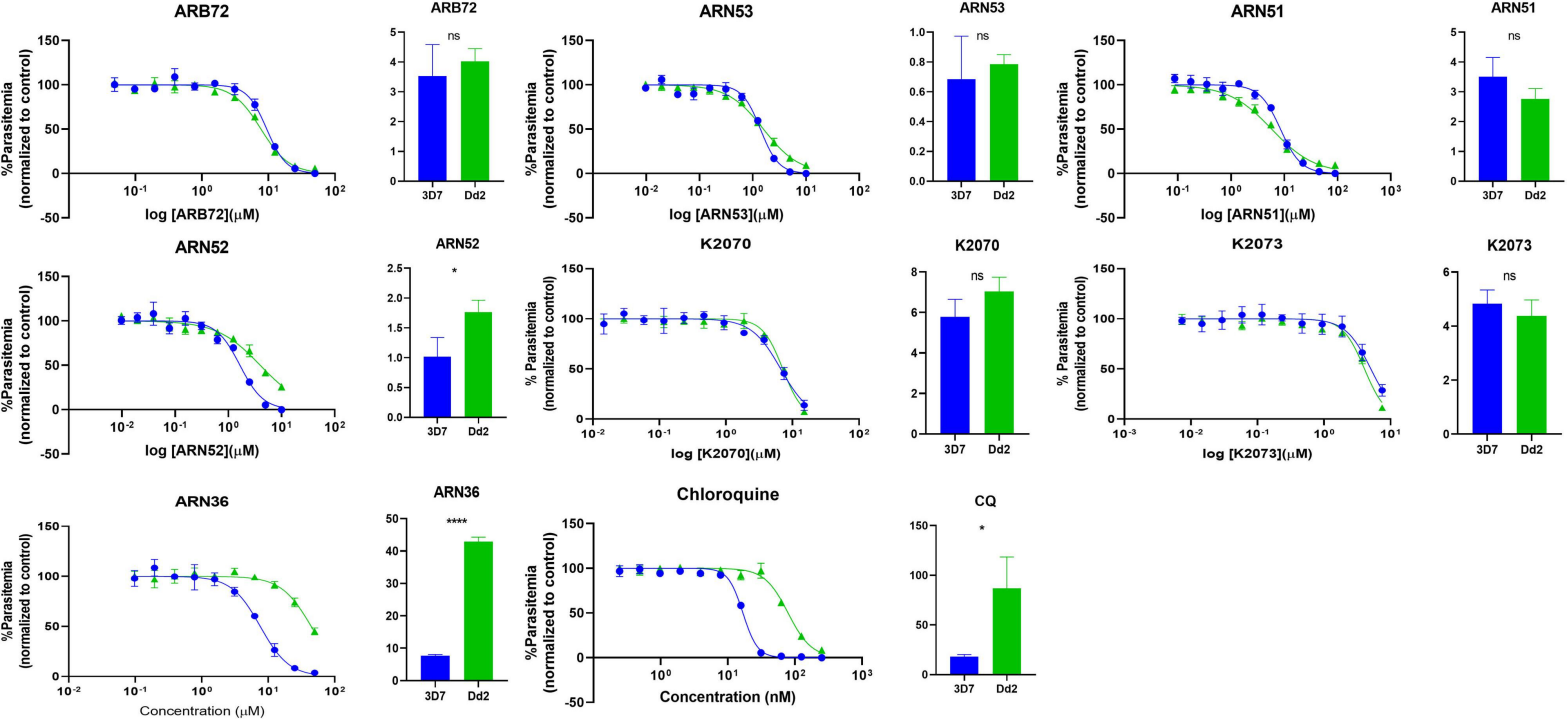
Comparative efficacy of the hybrid compounds against resistant *P. falciparum* Dd2 strain. Comparative inhibition curves and corresponding IC₅₀ histograms are shown for ARB72, ARN52, ARN53, ARN51, ARN36, K2070, and K2073 against the drug-sensitive 3D7 (blue) and drug-resistant Dd2 (green) strains, with chloroquine (CQ) included as a reference control. Parasite growth inhibition was assessed over 72 h under standard culture conditions, and IC₅₀ values were derived from nonlinear regression analysis. Comparative bar graphs summarize shifts in compound potency between strains, enabling evaluation of resistance-associated changes in activity. Statistical significance between 3D7 and Dd2 IC₅₀ values was determined using an unpaired *t*-test and is indicated as follows: *P < 0.05; ****P < 0.0001; ns, not significant. Data are presented as mean ± S.D. from three independent experiments, each performed in triplicate.

Our results revealed that two compounds, ARN52 and ARN36, showed statistically significant differences in activity between the two strains (*P* < 0.05) (Fig. 4). However, this difference reflected a loss of efficacy in the resistant Dd2 strain, suggesting that these compounds are susceptible to parasite resistance mechanisms. This implies that ARN52 and ARN36 may not be suitable as leads for overcoming resistance, although further mechanistic studies could provide valuable insights into how the parasite neutralizes their activity. One compound, ARN51, showed favorable activity based on the resistance index shown in Table 3 (R.I. = IC₅₀^Dd2^/IC₅₀^3D7^), with R.I. < 1 indicating enhanced potency against the resistant Dd2 strain. However, comparison between strains using an unpaired *t*-test did not reveal statistically significant differences. The remaining four compounds, by contrast, maintained their potency across both strains, underscoring their promise as candidates for further development.

### Compound ARN53 displayed gametocytocidal activity

Screening indole derivatives for antimalarial activity against the asexual stages of *Plasmodium* revealed varying levels of efficacy, with ARN53 emerging as the most promising candidate. Given its potent activity, ARN53 was selected for further evaluation against the parasite’s sexual stages. To assess its gametocytocidal activity, a modified NF54 *Plasmodium falciparum* strain engineered to express a luciferase reporter was employed. Late-stage gametocytes (stages IV and V) were incubated with five serially diluted concentrations of ARN53 for 72 h. Following incubation, cell viability was determined by measuring the luciferase signal, which is proportional to parasite survival. The assay involved lysing the cells and mixing the lysate with a luciferin substrate, enabling quantification of the luminescence signal. Gametocytocidal activity was expressed as the inverse of percent survival relative to the negative control (DMSO).

**Fig 5:**
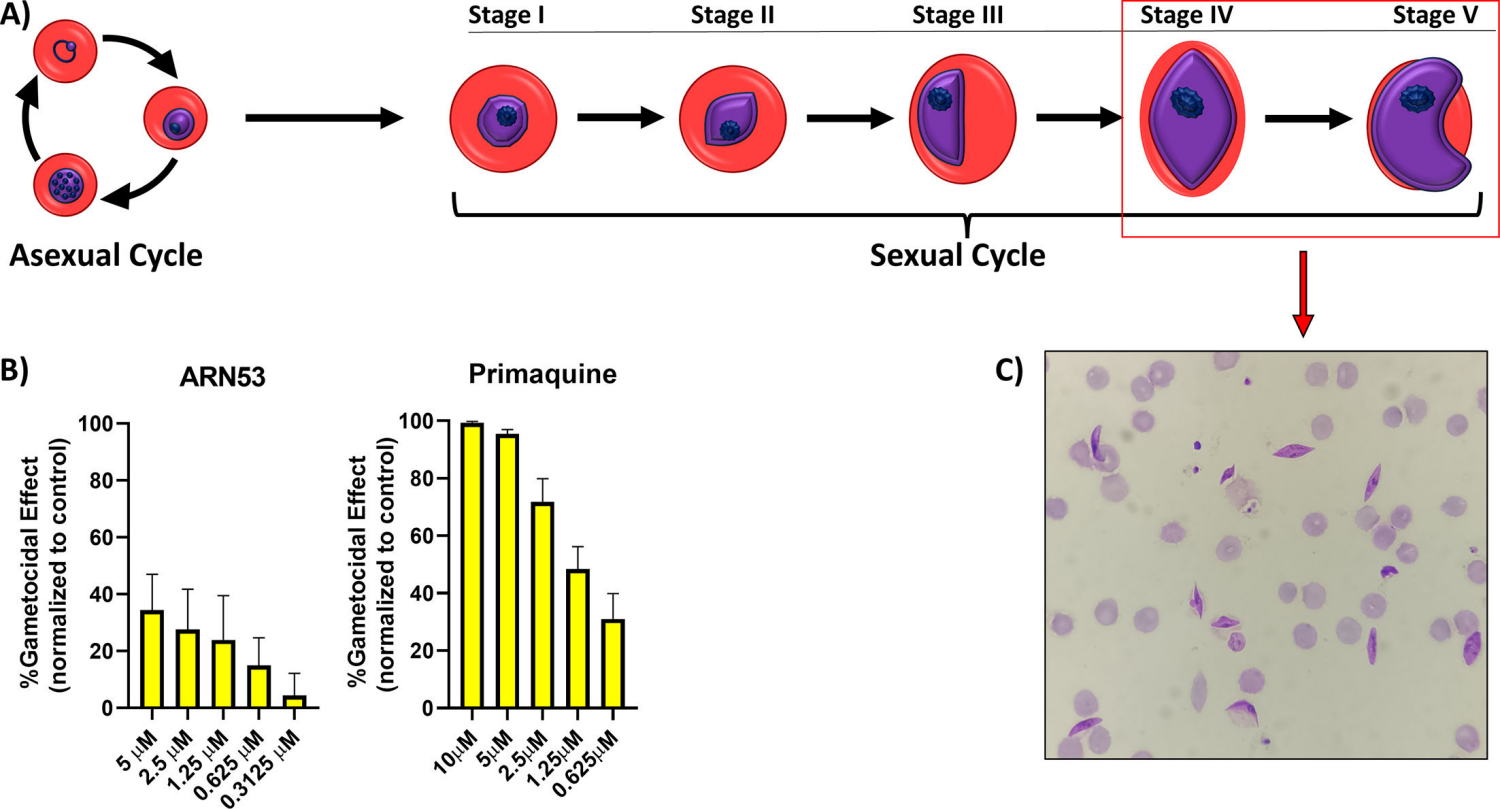
Gametocytocidal activity of ARN53 against late-stage *Plasmodium falciparum* gametocytes. (**A**) Schematic representation of the *P. falciparum* intraerythrocytic life cycle, highlighting the transition from asexual replication to sexual commitment and subsequent maturation into stage I–V gametocytes. (**B**) Histograms illustrating the inhibitory effect of ARN53 on mature stage IV–V gametocytes (left panel), in comparison with the reference drug primaquine (right panel). Late-stage NF54-GFP-luc gametocytes were treated with compounds for 72 h, and viability was assessed by luciferase activity. ARN53 exhibited a concentration-dependent reduction in gametocyte viability, enabling comparison of its transmission-blocking potential with that of established antimalarials. (**C**) Representative Panoptico-stained light microscopy image of untreated stage IV–V gametocytes, showing characteristic elongated morphology within erythrocytes prior to drug exposure. Images were acquired to confirm stage-specific enrichment and morphological integrity before treatment. Data are presented as mean ± S.D. from three independent experiments, each performed in triplicate.

Our results demonstrate that ARN53 exhibits an average gametocytocidal effect of 34.4% at its highest tested concentration of 5 µM (Fig. 5). A concentration-dependent decline in activity was observed as the compound was serially diluted in the subsequent wells. Interestingly, when compared with the positive control primaquine, there was substantial variability in ARN53’s effect, with maximum inhibition reaching 46%. In contrast, the well-established gametocytocidal agent exhibited minimal variability and potent activity across all replicates. This discrepancy suggests that ARN53 may exhibit stage-specific preference within the heterogeneous gametocyte population used in the assay, whereas primaquine targets gametocytes at all developmental stages without bias.

### Compound ARN53 synergizes with lumefantrine in combination

Compound ARN53, the most potent candidate among the tested series, was further evaluated for its interactions with established antimalarials currently used in treatment. A fixed-ratio isobologram method was employed, in which ARN53 was combined with chloroquine, primaquine, and lumefantrine at fixed ratios of 4:1, 3:2, 1:1, 2:3, and 1:4 to determine whether its mechanism of action complements or antagonizes those of the known drugs.

The ∑FIC₅₀ values obtained (Table 4) revealed that ARN53 interacts antagonistically with chloroquine and primaquine, with FIC₅₀ values ranging from 1.16 to 1.78 for chloroquine and 1.27 to 1.54 for primaquine. Interestingly, ARN53 showed a synergistic effect when combined with lumefantrine, with FIC₅₀ values ranging from 0.68 to 0.89. The strongest potentiation was observed when ARN53 was combined with lumefantrine at a 4:1 ratio.

**TABLE 4:** Interaction profiles of ARN53 in combination with reference antimalarial drugs against *Plasmodium falciparum* 3D7^*a*^

Combination	FIC_50_ ± S.D.					∑FIC_50_ ± S.D.
	4:1	3:2	1:1	2:3	1:4	
ARN53 + CQ	1.78 ± 0.1	1.58 ± 0.08	1.49 ± 0.09	1.44 ± 0.07	1.16 ± 0.08	1.49 ± 0.05
ARN53 + PMQ	1.47 ± 0.09	1.54 ± 0.07	1.46 ± 0.23	1.35 ± 0.11	1.27 ± 0.08	1.42 ± 0.07
ARN53 + LF	0.68 ± 0.09	0.7 ± 0.23	0.77 ± 0.28	0.75 ± 0.15	0.89 ± 0.43	0.76 ± 0.17

^*a*^ Drug interactions were evaluated using fixed-ratio combination assays, and fractional inhibitory concentration (FIC₅₀) values were determined at different volumetric ratios (4:1, 3:2, 1:1, 2:3, and 1:4). Data are presented as mean ± standard deviation (S.D.) from three independent experiments performed in triplicate. The sum of FIC₅₀ values (∑FIC₅₀) was calculated for each combination to assess the overall interaction profile. Interaction profiles were interpreted based on ΣFIC₅₀ values, where ΣFIC₅₀ < 1 indicates synergism, ΣFIC₅₀ ≈ 1 indicates an additive effect, and ΣFIC₅₀ > 1 indicates antagonism.

**Fig 6:**
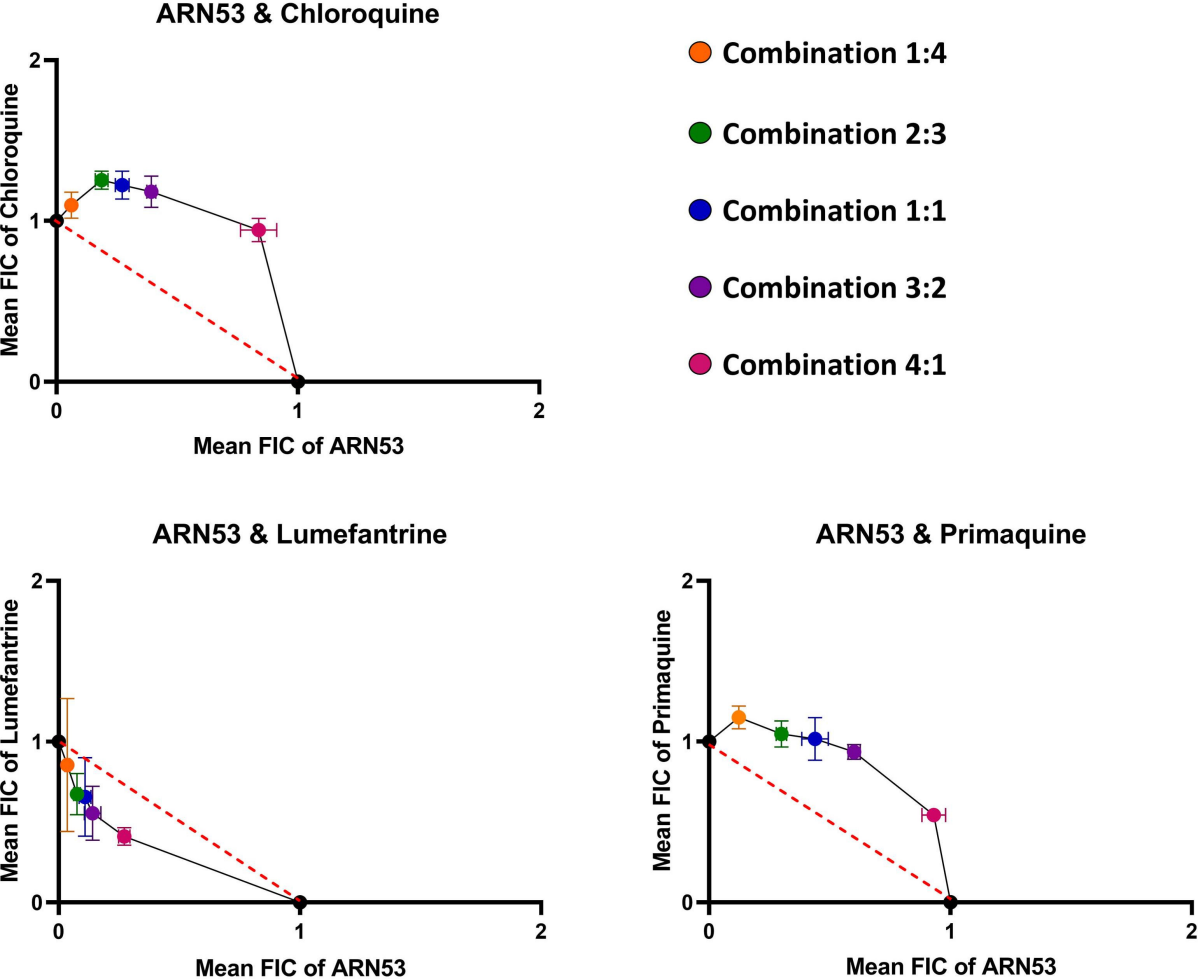
Drug interaction profiles of ARN53 in combination with standard antimalarials. Isobologram analysis was performed to evaluate interactions between ARN53 and standard antimalarial drugs (chloroquine, lumefantrine, and primaquine) at fixed ratios (4:1, 3:2, 1:1, 2:3, and 1:4). Fractional inhibitory concentration (FIC₅₀) values were calculated from IC₅₀ measurements obtained for each drug in combination relative to when tested alone and were plotted to generate isobolograms. The sum of FIC₅₀ values (ΣFIC₅₀) was used to assess interaction profiles, with data points positioned relative to the reference line of additivity (red dashed line). Points below the line indicate synergistic interactions, those on or near the line indicate additive effects, and those above the line indicate antagonism. Data are presented as mean ± S.D. from three independent experiments in triplicate.

The isobolograms in Fig. 6 illustrate these interactions: convex curves for chloroquine-primaquine combinations and a concave curve for lumefantrine, providing a clear visualization of antagonistic and synergistic effects, respectively.

## DISCUSSION

Malaria remains one of the most persistent global health challenges, and the emergence of partial resistance to artemisinin-based combination therapies emphasizes the need for new chemotypes with a distinct mechanism of action. An ideal antimalarial drug candidate should target multiple stages of the *Plasmodium* life cycle while also bypassing well-guarded resistance pathways. The present study contributes to this objective by identifying a class of indole-based hybrid compounds with potent and selective activity against *Plasmodium falciparum*.

We began by evaluating a collection of 24 hybrid compounds for their ability to inhibit parasite growth. We observed nine compounds exhibiting activity in the lower micromolar range, with ARN53 emerging as the most potent analog (IC₅₀ in the nanomolar range). Compounds belonging to the canonical, unprotected tryptophan-containing peptide series were inactive, indicating that this scaffold is unlikely to provide a productive direction for future antimalarial development thus far, as their natural peptidic sequences are prone to rapid enzymatic digestion, resulting in no activity. In contrast, within the noncanonical, protected, C-2-peripherally modified tryptophan-containing peptides, there was potential to direct endeavors toward antimalarial development. These peptide series contain an unnatural indole skeleton tethered to a monoterpenoid unit at the C-2 position, thereby conferring enhanced enzymatic resistance. As a result, within this series, the ARN53 compound was identified as the most promising antimalarial candidate. Interestingly, aside from the functionalization at the C-2 position, this compound incorporates an additional peripheric Csp^2^-N bond as a pyrimidine atom appended to the indole skeleton. The improved IC_50_ value (ca. 1,000-fold more potent) for ARN53 among its analogs highlighted a crucial *N*_(in)_-heteroaryl scaffold feature that could be straightforwardly envisioned in the next generation of antimalarials. On the other hand, when the indole ring is fused to functionalized tetrahydroquinoline frameworks, activity declines, as observed for the three compounds in the third series. Certainly, a pattern in activity is noticed, wherein the less peripherally substituted indole skeletons display the less encouraging results. Arguably, from a synthetic perspective, the unique advantage of combining C-2 and N(in) modifications, as exemplified by the ARN53 compound, lies in its skeletal arrangement, which significantly enhances its efficacy relative to its counterparts. Cytotoxicity profiling (in HEK293T cells) confirmed that active derivatives, including ARN53, displayed high parasite selectivity, whereas inactive or moderately active compounds, such as ARL35, exhibited host-cell toxicity. This correlation indicates that the observed cytotoxic phenotype was parasite-specific and linked to structural determinants of potency within the indole series.

To assess whether these compounds could overcome classical resistance, we evaluated their activity against the Dd2 strain. This strain is particularly relevant because it carries resistance to multiple frontline drugs, including chloroquine and SP. While chloroquine remains an important treatment for *P. vivax* malaria in endemic regions, it is no longer recommended for *P. falciparum* because of widespread resistance (48). Likewise, increasing resistance to SP continues to compromise the efficacy of intermittent preventive treatment in pregnancy (IPTp-SP), a key strategy for protecting pregnant women and infants in malaria-endemic areas (49). More broadly, resistance to antifolate drugs targeting dihydrofolate reductase (DHFR), such as pyrimethamine, has intensified the search for new therapeutic options, including next-generation antifolates like P218 (50) and repurposed drugs such as Duloxetine and Guanethidine (51). Artemisinin-based combination therapies (ACTs) were originally introduced to overcome resistance to earlier antimalarial regimens; however, increasing reports of partial artemisinin resistance further underscore the need for new effective compounds. In this context, Dd2 is a valuable platform for identifying candidates capable of overcoming current resistance barriers and restoring therapeutic efficacy.

Most of the active derivatives retained comparable potency against both sensitive and resistant parasites. This finding suggested that their mode of action was independent of the pathways mediating resistance, especially chloroquine. As expected, chloroquine itself showed loss of activity in Dd2, serving as a reference control. In contrast, ARN36 showed a marked decrease in potency in the resistant strain, highlighting how subtle structural variations within this series can profoundly affect both mechanism and resistance susceptibility. From these observations, we reasoned that ARN36-like scaffolds may not be suitable for further development, while ARN53 remained the most reliable candidate.

A persistent limitation of current antimalarial therapies is the scarcity of agents that effectively target gametocytes—the sexual stage of *Plasmodium* responsible for transmission to mosquitoes. Only a few compounds exhibit measurable activity at this stage, and primaquine remains the gold standard despite its modest micromolar efficacy and rapid systemic clearance (52). To assess whether ARN53 could help overcome this challenge, we evaluated its gametocytocidal potential using a luciferase-expressing NF54 strain, in which gametocyte viability correlates directly with luminescence. ARN53 showed measurable activity against gametocytes, although with considerable variability. This inconsistency may indicate stage-specific effects on mature gametocytes, particularly given that the assay used does not distinguish between stage IV and stage V populations and instead relies on a heterogeneous mixture. In contrast, its nanomolar potency in asexual-stage assays suggests that its primary mode of action targets pathways more critical for the erythrocytic cycle than for gametocyte metabolism or transmission. While this introduces some uncertainty about its precise mechanism, it also offers a useful starting point for further structural optimization to enhance selectivity for transmission-blocking activity.

Given the established importance of combination therapy in delaying resistance, we expanded our study to investigate the behavior of ARN53 in the presence of already established antimalarials. Combination regimens are central to sustained malaria control, and a clear understanding of drug–drug interactions is essential for rational therapeutic design. Using fixed-ratio isobologram analysis, ARN53 displayed antagonistic interactions with chloroquine and primaquine and a synergistic interaction with lumefantrine.

The rationale for including chloroquine comes from recent studies suggesting that it may still have value as a partner drug in triple artemisinin-based combination therapies. In experimental malaria models, adding chloroquine to standard ACT improved parasite clearance and delayed recrudescence, supporting its potential as a complementary antimalarial partner (9). Primaquine was also included in the combination analysis because of its established role in transmission blocking and its potential to complement blood-stage antimalarials in a combined therapeutic regimen (53). Integrating primaquine with compounds active against asexual stages may reduce the need for separate administration while broadening treatment efficacy through both parasite clearance and gametocyte targeting. In addition, previous studies have reported synergistic interactions between primaquine and established antimalarials (54), making it a useful reference for assessing the interaction profile and therapeutic potential of ARN53.

Lumefantrine is a lipophilic aryl-amino alcohol widely used in combination with artemether as a first-line treatment for *Plasmodium falciparum* malaria (55). Owing to its prolonged half-life, lumefantrine contributes to the clearance of residual blood-stage parasites in combination therapy, thereby preventing recrudescence (56). The drug is primarily active against erythrocytic stages, with minimal activity against sexual forms (57). Although its precise mechanism remains incompletely defined, lumefantrine is thought to act within the parasite’s food vacuole by inhibiting heme detoxification, resulting in the accumulation of toxic free heme and subsequent parasite death (58).

The observed synergy in our study suggests that ARN53 and lumefantrine act through distinct yet complementary mechanisms, resulting in greater efficacy than either compound alone. Notably, lumefantrine retains activity against chloroquine-resistant *Plasmodium falciparum* strains, as *pfcrt* mutations do not reduce lumefantrine susceptibility and may even enhance activity through altered drug distribution within the parasite (59). In contrast, lumefantrine treatment consistently selects for specific *pfmdr1* alleles and increased gene copy number, indicating that its activity is influenced by transporter-mediated processes associated with the digestive vacuole (60). Concurrently, hexahydroquinolines were shown to disrupt hemoglobin uptake and reduce hemozoin formation while exhibiting *pfmdr1*-dependent resistance, and parasites resistant to hexahydroquinolines displayed reciprocal sensitivity to lumefantrine (60). Hence, all evidence supports lumefantrine’s mode of action to be distinct from chloroquine, which accumulates within the digestive vacuole as a weak base following protonation (61). Instead, lumefantrine appears to act through parasite heme-associated pathways influenced by the digestive vacuole transport dynamics.

It was also important to investigate whether our lead compound interferes with the activity of established antimalarials when used in combination, particularly with transmission-blocking gametocytocidal drugs such as primaquine. Combining two drugs offers little therapeutic benefit if they act against each other and ultimately reduce efficacy. Hence, we evaluated ARN53 in combination with both chloroquine and primaquine. The antagonistic interactions observed with these compounds reinforce our earlier findings that, although most compounds in this series retain activity by bypassing parasite survival strategies, combining ARN53 with chloroquine or primaquine may result in unfavorable therapeutic outcomes. Furthermore, as established by previous work (54), the absence of synergy between ARN53 and primaquine may indicate that ARN53 operates through a pathway completely distinct from that of conventional blood-stage schizonticidal antimalarials. Such a mode of action could explain its retained activity against resistant strains and highlights the need for further studies to elucidate its molecular target and assess its therapeutic potential. In this context, the retained activity of ARN53 against the multidrug-resistant Dd2 strain observed in this study, together with its synergistic interaction with lumefantrine, suggests that ARN53 may target complementary aspects of heme metabolism or digestive vacuole function. Rather than acting within the same intravacuolar space as chloroquine or interfering with folate biosynthesis as SP, ARN53 may act upstream of or at the interface of these pathways, thereby enhancing lumefantrine efficacy by further perturbing heme detoxification or related processes. Our findings conclude that lumefantrine could serve as a valuable partner for ARN53 in future antimalarial formulations.

Taken together, our study identifies ARN53 as a potent, selective, and resistance-resilient antimalarial lead. The compound’s promising gametocytocidal activity, along with its strong performance against blood-stage parasites and its synergistic interaction with lumefantrine, presents avenues for further development. Overall, these findings expand the chemical and pharmacophoric space accessible to indole scaffolds, contributing to the continued development of next-generation antimalarial agents capable of overcoming multidrug resistance.

## SUPPLEMENTAL MATERIAL

Supplemental material aac.00490-26-s0001.docx Fig. S1.

ASM does not own the copyrights to Supplemental Material that may be linked to, or accessed through, an article. The authors have granted ASM a non-exclusive, world-wide license to publish the Supplemental Material files. Please contact the corresponding author directly for reuse.

## References

[1] Klepac P, Hsieh JL, Ducker CL, Assoum M, Booth M, Byrne I, Dodson S, Martin DL, Turner CMR, van Daalen KR, et al.. 2024. Climate change, malaria and neglected tropical diseases: a scoping review. Trans R Soc Trop Med Hyg 118:561–579. doi:10.1093/trstmh/trae026 38724044 PMC11367761

[2] González-Sanz M, Berzosa P, Norman FF. 2023. Updates on malaria epidemiology and prevention strategies. Curr Infect Dis Rep:1–9. doi:10.1007/s11908-023-00805-9 PMC10248987 37361492

[3] Boni MF. 2022. Breaking the cycle of malaria treatment failure. Front Epidemiol 2:1041896. doi:10.3389/fepid.2022.1041896 38455307 PMC10910953

[4] Hanboonkunupakarn B, White NJ. 2022. Advances and roadblocks in the treatment of malaria. Br J Clin Pharmacol 88:374–382. doi:10.1111/bcp.14474 32656850 PMC9437935

[5] Hernandez Maldonado J, Grundmann O. 2022. Drug-drug interactions of artemisinin-based combination therapies in malaria treatment: a narrative review of the literature. J Clin Pharmacol 62:1197–1205. doi:10.1002/jcph.2073 35543380

[6] Kokori E, Olatunji G, Akinboade A, Akinoso A, Egbunu E, Aremu SA, Okafor CE, Oluwole O, Aderinto N. 2024. Triple artemisinin-based combination therapy (TACT): advancing malaria control and eradication efforts. Malar J 23:25. doi:10.1186/s12936-024-04844-y 38238781 PMC10797909

[7] World Health Organization. 2023. WHO guidelines for malaria, 14 March 2023. World Health Organization.

[8] Nguyen TD, Gao B, Amaratunga C, Dhorda M, Tran TN-A, White NJ, Dondorp AM, Boni MF, Aguas R. 2023. Preventing antimalarial drug resistance with triple artemisinin-based combination therapies. Nat Commun 14:4568. doi:10.1038/s41467-023-39914-3 37516752 PMC10387089

[9] Akinola O, Afolabi OO, Adebisi-Jose GO, Amusan AI, Olumoh-Adbul HA, Olabanji O, Teslim O, Gbotosho GO. 2025. Triple artemisinin-based combination therapy (TACT): efficacy of dihydroartemisinin-piperaquine plus chloroquine against Plasmodium berghei ANKA strains with different drug sensitivities in a murine malaria model. J Infect Chemother 31:102556. doi:10.1016/j.jiac.2024.11.006 39536985

[10] Ward KE, Fidock DA, Bridgford JL. 2022. Plasmodium falciparum resistance to artemisinin-based combination therapies. Curr Opin Microbiol 69:102193. doi:10.1016/j.mib.2022.102193 36007459 PMC9847095

[11] Bassat Q, Maïga-Ascofaré O, May J, Clain J, Mombo-Ngoma G, Groger M, Adegnika AA, Agobé J-CD, Djimde A, Mischlinger J, Ramharter M, ASAAP and Multimal Consortia. 2022. Challenges in the clinical development pathway for triple and multiple drug combinations in the treatment of uncomplicated falciparum malaria. Malar J 21:61. doi:10.1186/s12936-022-04079-9 35193586 PMC8864855

[12] Pandit K, Surolia N, Bhattacharjee S, Karmodiya K. 2023. The many paths to artemisinin resistance in Plasmodium falciparum. Trends Parasitol 39:1060–1073. doi:10.1016/j.pt.2023.09.011 37833166

[13] World Health Organization. 2025. Malaria surveillance, monitoring and evaluation: a reference manual. World Health Organization.

[14] Motta FC, McGoff K, Moseley RC, Cho CY, Kelliher CM, Smith LM, Ortiz MS, Leman AR, Campione SA, Devos N, Chaorattanakawee S, Uthaimongkol N, Kuntawunginn W, Thongpiam C, Thamnurak C, Arsanok M, Wojnarski M, Vanchayangkul P, Boonyalai N, Smith PL, Spring MD, Jongsakul K, Chuang I, Harer J, Haase SB. 2023. The parasite intraerythrocytic cycle and human circadian cycle are coupled during malaria infection. Proc Natl Acad Sci USA 120:e2216522120. doi:10.1073/pnas.2216522120 37279274 PMC10268210

[15] Hunter FK, Butler TD, Gibbs JE. 2022. Circadian rhythms in immunity and host-parasite interactions. Parasite Immunol 44:e12904. doi:10.1111/pim.12904 34971451 PMC9285061

[16] Hotta CT, Gazarini ML, Beraldo FH, Varotti FP, Lopes C, Markus RP, Pozzan T, Garcia CR. 2000. Calcium-dependent modulation by melatonin of the circadian rhythm in malarial parasites. Nat Cell Biol 2:466–468. doi:10.1038/35017112 10878815

[17] Beraldo FH, Almeida FM, da Silva AM, Garcia CRS. 2005. Cyclic AMP and calcium interplay as second messengers in melatonin-dependent regulation of Plasmodium falciparum cell cycle. J Cell Biol 170:551–557. doi:10.1083/jcb.200505117 16103224 PMC2171486

[18] Alves E, Bartlett PJ, Garcia CRS, Thomas AP. 2011. Melatonin and IP3-induced Ca2+ release from intracellular stores in the malaria parasite Plasmodium falciparum within infected red blood cells. J Biol Chem 286:5905–5912. doi:10.1074/jbc.M110.188474 21149448 PMC3037703

[19] Koyama FC, Ribeiro RY, Garcia JL, Azevedo MF, Chakrabarti D, Garcia CRS. 2012. Ubiquitin proteasome system and the atypical kinase PfPK7 are involved in melatonin signaling in Plasmodium falciparum. J Pineal Res 53:147–153. doi:10.1111/j.1600-079X.2012.00981.x 22348509 PMC3360131

[20] Gazarini ML, Beraldo FH, Almeida FM, Bootman M, Da Silva AM, Garcia CRS. 2011. Melatonin triggers PKA activation in the rodent malaria parasite Plasmodium chabaudi. J Pineal Res 50:64–70. doi:10.1111/j.1600-079X.2010.00810.x 20964707

[21] Dias BKM, Nakabashi M, Alves MRR, Portella DP, Dos Santos BM, Costa da Silva Almeida F, Ribeiro RY, Schuck DC, Jordão AK, Garcia CRS. 2020. The Plasmodium falciparum eIK1 kinase (PfeIK1) is central for melatonin synchronization in the human malaria parasite. Melatotosil blocks melatonin action on parasite cell cycle. J Pineal Res 69:e12685. doi:10.1111/jpi.12685 32702775 PMC7539967

[22] Singh MK, Bonnell VA, Tojal Da Silva I, Santiago VF, Moraes MS, Adderley J, Doerig C, Palmisano G, Llinas M, Garcia CRS. 2024. A Plasmodium falciparum MORC protein complex modulates epigenetic control of gene expression through interaction with heterochromatin. eLife 12:RP92201. doi:10.7554/eLife.92201 39412522 PMC11483127

[23] Scarpelli PH, Tessarin-Almeida G, Viçoso KL, Lima WR, Borges-Pereira L, Meissner KA, Wrenger C, Raffaello A, Rizzuto R, Pozzan T, Garcia CRS. 2019. Melatonin activates FIS1, DYN1, and DYN2 Plasmodium falciparum related-genes for mitochondria fission: Mitoemerald-GFP as a tool to visualize mitochondria structure. J Pineal Res 66:e12484. doi:10.1111/jpi.12484 29480948 PMC6585791

[24] Koyama FC, Azevedo MF, Budu A, Chakrabarti D, Garcia CRS. 2014. Melatonin-induced temporal up-regulation of gene expression related to ubiquitin/proteasome system (UPS) in the human malaria parasite Plasmodium falciparum. Int J Mol Sci 15:22320–22330. doi:10.3390/ijms151222320 25479077 PMC4284710

[25] Dias B. K. M., Mohanty A, Garcia CRS. 2024. Melatonin as a circadian marker for Plasmodium rhythms. Int J Mol Sci 25:7815. doi:10.3390/ijms25147815 39063057 PMC11277106

[26] Schuck DC, Jordão AK, Nakabashi M, Cunha AC, Ferreira VF, Garcia CRS. 2014. Synthetic indole and melatonin derivatives exhibit antimalarial activity on the cell cycle of the human malaria parasite Plasmodium falciparum. Eur J Med Chem 78:375–382. doi:10.1016/j.ejmech.2014.03.055 24699367

[27] Surur AS, Huluka SA, Mitku ML, Asres K. 2020. Indole: the after next scaffold of antiplasmodial agents? Drug Des Devel Ther 14:4855–4867. doi:10.2147/DDDT.S278588 PMC7666986 33204071

[28] Dhameliya TM, Vekariya DD, Bhatt PR, Kachroo T, Virani KD, Patel KR, Bhatt S, Dholakia SP. 2025. Synthetic account on indoles and their analogues as potential anti-plasmodial agents. Mol Divers 29:871–897. doi:10.1007/s11030-024-10842-8 38709459

[29] Lunga MJ, Chisango RL, Weyers C, Isaacs M, Taylor D, Edkins AL, Khanye SD, Hoppe HC, Veale CGL. 2018. Expanding the SAR of nontoxic antiplasmodial indolyl-3-ethanone ethers and thioethers. ChemMedChem 13:1353–1362. doi:10.1002/cmdc.201800235 29756273

[30] Luthra T, Nayak AK, Bose S, Chakrabarti S, Gupta A, Sen S. 2019. Indole based antimalarial compounds targeting the melatonin pathway: their design, synthesis and biological evaluation. Eur J Med Chem 168:11–27. doi:10.1016/j.ejmech.2019.02.019 30798050

[31] Mallaupoma LRC, Dias BK de M, Singh MK, Honorio RI, Nakabashi M, Kisukuri C de M, Paixão MW, Garcia CRS. 2022. Decoding the role of melatonin structure on Plasmodium falciparum human malaria parasites synchronization using 2-sulfenylindoles derivatives. Biomolecules 12:638. doi:10.3390/biom12050638 35625565 PMC9138683

[32] Mohanty A, Carlos L, Barbosa EG, Jordão AK, Garcia C. 2025. Targeting Plasmodium falciparum asexual and sexual stages with melatonin‐based indole compounds. ChemistrySelect 10:e04720. doi:10.1002/slct.202504720

[33] Pacheco PAF, Santos MMM. 2022. Recent progress in the development of indole-based compounds active against malaria, trypanosomiasis and leishmaniasis. Molecules 27:1. doi:10.3390/molecules27010319 PMC8746838 35011552

[34] da Silva W, Torres T, Barros de Menezes RP, Scotti MT, Reimão JQ, Duarte Correia CR. 2025. Novel indole-based marinoquinolines as promising candidates against Toxoplasma gondii. Eur J Med Chem 299:118057. doi:10.1016/j.ejmech.2025.118057 40812069

[35] Abdelhalim WA, Rabee AR, Soliman SM, Hagar M, Moneer EA, Bakr BA, Barakat A, Haukka M, Rasheed HA. 2025. New formyl indole derivatives based on thiobarbituric acid and their nano-formulations; synthesis, characterization, parasitology and histopathology investigations. Sci Rep 15:299. doi:10.1038/s41598-024-81683-6 39747136 PMC11696224

[36] Funkhouser-Jones LJ, Xu R, Wilke G, Fu Y, Schriefer LA, Makimaa H, Rodgers R, Kennedy EA, VanDussen KL, Stappenbeck TS, Baldridge MT, Sibley LD. 2023. Microbiota-produced indole metabolites disrupt mitochondrial function and inhibit Cryptosporidium parvum growth. Cell Rep 42:112680. doi:10.1016/j.celrep.2023.112680 37384526 PMC10530208

[37] Chappell CL, Darkoh C, Shimmin L, Farhana N, Kim DK, Okhuysen PC, Hixson J. 2016. Fecal indole as a biomarker of susceptibility to Cryptosporidium infection. Infect Immun 84:2299–2306. doi:10.1128/IAI.00336-16 27245413 PMC4962629

[38] Fivelman QL, Adagu IS, Warhurst DC. 2004. Modified fixed-ratio isobologram method for studying in vitro interactions between atovaquone and proguanil or dihydroartemisinin against drug-resistant strains of Plasmodium falciparum. Antimicrob Agents Chemother 48:4097–4102. doi:10.1128/AAC.48.11.4097-4102.2004 15504827 PMC525430

[39] Li X, He Z, Chen L, Li Y, Li Q, Zhao S, Tao Z, Hu W, Qin L, Chen X. 2011. Synergy of the antiretroviral protease inhibitor indinavir and chloroquine against malaria parasites in vitro and in vivo. Parasitol Res 109:1519–1524. doi:10.1007/s00436-011-2427-z 21537980

[40] Rajendran V, Gurukkalot KJF i. DD. 2023. In vitro drug interaction of ionophores with artemisinin and chloroquine against Plasmodium falciparum 3D7 blood-stage infection. Front Drug Discov 3:1257698. doi:10.3389/fddsv.2023.1257698

[41] Lima RN, Delgado JAC, Bernardi DI, Berlinck RGS, Kaplaneris N, Ackermann L, Paixão MW. 2021. Post-synthetic functionalization of tryptophan protected peptide sequences through indole (C-2) photocatalytic alkylation. Chem Commun (Camb) 57:5758–5761. doi:10.1039/d1cc01822a 34002741

[42] Delgado JAC, Tian YM, Marcon M, Konig B, Paixao MW. 2023. Side-selective solid-phase metallaphotoredox N(_in_)-arylation of peptides. J Am Chem Soc 145:26452–26462. doi:10.1021/jacs.3c10792 37976043

[43] de Souza Quaglio K. 2021. Desenvolvimento de metodologias sustentáveis para a síntese de tetraidroquinolinas: o uso de hemiacetais como indutores de assimetria. Universidade Federal de São Carlos

[44] Sidhu ABS, Verdier-Pinard D, Fidock DA. 2002. Chloroquine resistance in Plasmodium falciparum malaria parasites conferred by pfcrt mutations. Science 298:210–213. doi:10.1126/science.1074045 12364805 PMC2954758

[45] Baniecki ML, Wirth DF, Clardy J. 2007. High-throughput Plasmodium falciparum growth assay for malaria drug discovery. Antimicrob Agents Chemother 51:716–723. doi:10.1128/AAC.01144-06 17116676 PMC1797774

[46] Wang P, Read M, Sims PF, Hyde JE. 1997. Sulfadoxine resistance in the human malaria parasite Plasmodium falciparum is determined by mutations in dihydropteroate synthetase and an additional factor associated with folate utilization. Mol Microbiol 23:979–986. doi:10.1046/j.1365-2958.1997.2821646.x 9076734

[47] Masserey T, Braunack-Mayer L, Miller RS, Möhrle JJ, Penny MA. 2025. A roadmap for understanding sulfadoxine-pyrimethamine in malaria chemoprevention. Parasitology 152:133–142. doi:10.1017/S0031182025000071 39844654 PMC12089447

[48] Organization, W. H. 2025. WHO guidelines for malaria, 13 august 2025

[49] Sundararaman SA, Odom John AR. 2022. Prevention of malaria in pregnancy: the threat of sulfadoxine-pyrimethamine resistance. Front Pediatr 10:966402. doi:10.3389/fped.2022.966402 36061376 PMC9433640

[50] Pethrak C, Posayapisit N, Pengon J, Suwanakitti N, Saeung A, Shorum M, Aupalee K, Taai K, Yuthavong Y, Kamchonwongpaisan S, Jupatanakul N. 2022. New insights into antimalarial chemopreventive activity of antifolates. Antimicrob Agents Chemother 66:e0153821. doi:10.1128/AAC.01538-21 34930029 PMC8846393

[51] Verma K, Chaturvedi R, Lahariya AK, Verma AK, Schneider KA, Anvikar AR, Bharti PK. 2024. Repurposing FDA-approved drugs to target malaria through inhibition of dihydrofolate reductase in the folate biosynthesis pathway: a prospective approach. J Cell Biochem 125:e30533. doi:10.1002/jcb.30533 38345373

[52] Yilma D, Stepniewska K, Bousema T, Drakeley C, Eachempati P, Guerin PJ, Mårtensson A, Mwaiswelo R, Taylor WR, Barnes KI, WWARN Paediatric Primaquine for P falciparum Transmission Blocking Study Group. 2025. Safety and efficacy of single-dose primaquine to interrupt Plasmodium falciparum malaria transmission in children compared with adults: a systematic review and individual patient data meta-analysis. Lancet Infect Dis 25:965–976. doi:10.1016/S1473-3099(25)00078-7 40286803 PMC12353880

[53] Pukrittayakamee S, Chotivanich K, Chantra A, Clemens R, Looareesuwan S, White NJ. 2004. Activities of artesunate and primaquine against asexual- and sexual-stage parasites in falciparum malaria. Antimicrob Agents Chemother 48:1329–1334. doi:10.1128/AAC.48.4.1329-1334.2004 15047537 PMC375327

[54] Cabrera M, Cui L. 2015. In vitro activities of primaquine-schizonticide combinations on asexual blood stages and gametocytes of Plasmodium falciparum. Antimicrob Agents Chemother 59:7650–7656. doi:10.1128/AAC.01948-15 26416869 PMC4649191

[55] Kokwaro G, Mwai L, Nzila A. 2007. Artemether/lumefantrine in the treatment of uncomplicated falciparum malaria. Expert Opin Pharmacother 8:75–94. doi:10.1517/14656566.8.1.75 17163809

[56] Schnyder JL, Bache BE, Hoogakker M, Van De Ruit M, Zonneveld R, Hermans SM, Stijnis C, Van Vugt M, Hänscheid T, Van Hest RM, Goorhuis A, de Jong HK, Grobusch MP. 2025. Malaria recrudescence after artemether-lumefantrine treatment in travellers- a hospital-based observational study and literature review. Trop Dis Travel Med Vaccines 11:19. doi:10.1186/s40794-025-00256-1 40619429 PMC12232715

[57] Kumsa T, Jima B, Tamiru G, Getu R, Melaku M, Tesfaye Y, Lindtjørn B, Massebo F. 2025. Efficacy and gametocyte carriage in Plasmodium falciparum cases treated with artemether-lumefantrine alone versus with single-dose primaquine in Ethiopia: a randomized controlled study. Acta Trop 270:107800. doi:10.1016/j.actatropica.2025.107800 40849034

[58] Combrinck JM, Mabotha TE, Ncokazi KK, Ambele MA, Taylor D, Smith PJ, Hoppe HC, Egan TJ. 2013. Insights into the role of heme in the mechanism of action of antimalarials. ACS Chem Biol 8:133–137. doi:10.1021/cb300454t 23043646 PMC3548943

[59] Shafik SH, Richards SN, Corry B, Martin RE. 2022. Mechanistic basis for multidrug resistance and collateral drug sensitivity conferred to the malaria parasite by polymorphisms in PfMDR1 and PfCRT. PLoS Biol 20:e3001616. doi:10.1371/journal.pbio.3001616 35507548 PMC9067703

[60] Vanaerschot M, Lucantoni L, Li T, Combrinck JM, Ruecker A, Kumar TRS, Rubiano K, Ferreira PE, Siciliano G, Gulati S, et al.. 2017. Hexahydroquinolines are antimalarial candidates with potent blood-stage and transmission-blocking activity. Nat Microbiol 2:1403–1414. doi:10.1038/s41564-017-0007-4 28808258 PMC5708124

[61] Ehlgen F, Pham JS, de Koning-Ward T, Cowman AF, Ralph SA. 2012. Investigation of the Plasmodium falciparum food vacuole through inducible expression of the chloroquine resistance transporter (PfCRT). PLoS One 7:e38781. doi:10.1371/journal.pone.0038781 22719945 PMC3374814

