# Supplementary material for: Indole-based hybrids target both asexual parasites and gametocytes of Plasmodium falciparum and synergize with lumefantrine
Source: Antimicrob Agents Chemother. 2026 Jul 1;70(8):e00490-26. doi: 10.1128/aac.00490-26 (PMC13436391; doi:10.1128/aac.00490-26)
Supplement: Supplementary file 1 [file aac.00490-26-s0001.docx]

**Figure S1—Screening for anti-plasmodial activity in synthetic indole hybrids in wild-type parasites (3D7).** The figure shows representative growth inhibition and survival curves of *Plasmodium falciparum* in response to all compounds evaluated in this study, together with reference antimalarial drugs including chloroquine, primaquine, and lumefantrine used as controls. Asynchronous cultures of the 3D7 strain were treated with increasing concentrations of each compound for 72 hours under standard conditions. Parasitemia was measured by flow cytometry after staining with SYBR Green I to detect parasite nucleic acids and MitoTracker to assess mitochondrial membrane potential, allowing identification of viable parasites within infected red blood cells. Curves were generated by comparing parasite survival to untreated controls in order to evaluate compound potency. All experiments were performed in three independent biological replicates, each with triplicate measurements, and error bars represent the standard deviation (SD).
